# Critical learning from industrial catalysis for nanocatalytic medicine

**DOI:** 10.1038/s41467-024-48319-9

**Published:** 2024-05-08

**Authors:** Zhaokui Jin, Lingdong Jiang, Qianjun He

**Affiliations:** 1 Medical Center on Aging, Ruijin Hospital; Shanghai Key Laboratory of Hydrogen Science & Center of Hydrogen Science, School of Materials Science and Engineering, Shanghai Jiao Tong University, Shanghai, 200240 China; 2 School of Biomedical Engineering, Guangzhou Medical University, Guangzhou, 510182 China; 3 College of Pharmacy, Shenzhen Technology University, Shenzhen, 518118 China

**Keywords:** Nanoparticles, Nanotechnology in cancer, Biomedical materials

## Abstract

Systematical and critical learning from industrial catalysis will bring inspiration for emerging nanocatalytic medicine, but the relevant knowledge is quite limited so far. In this review, we briefly summarize representative catalytic reactions and corresponding catalysts in industry, and then distinguish the similarities and differences in catalytic reactions between industrial and medical applications in support of critical learning, deep understanding, and rational designing of appropriate catalysts and catalytic reactions for various medical applications. Finally, we summarize/outlook the present and potential translation from industrial catalysis to nanocatalytic medicine. This review is expected to display a clear picture of nanocatalytic medicine evolution.

## Abstract (web summary)

The knowledge gained from industrial catalysis benefits advancements of nanocatalytic medicines. Here the authors review the similarities, differences and connections in catalytic reactions between industrial and medical applications to support deep understanding and rational design of nanocatalytic medicines.

## Introduction

Industrial catalysis has made great impact on the enhancement of chemicals production efficacy and quality. Aiming to a variety of emerging application fields such as biomedical applications, the development of advanced catalytic methodologies and specific catalysts is urgent, important but challenging. Just as inspired from the ingenuity of biological activities, the biocatalysis has been industrially developed for chemical synthesis. Conversely, learning from the abundant knowledge of industrial catalysis will provide inspiration for catalytic medicine, which is an emerging field only involving a small part of catalytic reactions and catalysts at present, implying the necessity of learning from industrial catalysis. As an early example, a Fenton-like catalytic reaction based on amorphous iron nanoparticles was developed for tumor microenvironment (H_2_O_2_/H^+^)-responsive catalytic generation of ∙OH to kill cancer cells in situ^1^. Subsequently, the concept of nanocatalytic medicine proposed by Shi incubates an emerging cross-cutting field of utilizing nanocatalytic approaches to resolve medical problems^1–5^. Noticeably, efficiently and selectively catalytic treatment of diseases can be harnessed to lower drug dosage and avoid the off-target toxicity-induced side effects. The hidden treasure in industrial catalysis is an ideal tool to fuel the future development of nanocatalytic medicine. For inspiring learning, we summarize general catalytic reactions and catalysts, especially those potentially valuable for medicine, in this review.

Reactants, desired products and catalytic reaction conditions of in vivo medical catalysis are much different from that of industrial catalysis. High temperature, pressure and acidity/alkalinity for efficient industrial catalysis are unsuitable to in vivo application, and thus optimal catalytic conditions and catalysts have to be modified for the same reaction. Especially, some special reactants and products in vivo are never involved in industrial catalysis, and thus these catalytic reactions and corresponding catalysts have to be reconsidered. Therefore, this review distinguishes the similarities and differences between industrial catalysis and medicine catalysis for better biomedical applications of catalysis.

Nanomedicines, which are generally constructed by nanocarrier encapsulating drug, are developed to improve the bioavailability of therapeutic and diagnostic agents. However, the severe off-target of nanomedicines is currently unavoidable owing to various complicated biological barriers, leaving the unresolved risk of toxicity and the limited theranostic efficacy. Totally different from direct drug delivery by nanomedicines, both drugs and varied therapeutic agents such as reactive oxygen species (ROS) and therapeutic gas molecules can be locally synthesized by catalysis, and even catalytic consumption of reactants can also make contribution to therapeutic outcome. Selectivity, specificity, localization and acceleration from catalytic nanomedicines can not only greatly enhance therapeutic outcomes, but also mitigate the risk of off-target mediated toxicity. Therefore, this review demonstrates the strategies of catalytically improving therapeutic efficacy. Finally, we lay out current challenges and future directions of nanocatalytic medicine development with a view of maximizing its great potential.

## Inspiring learning: general catalytic reactions and catalysts

**Fig. 1:**
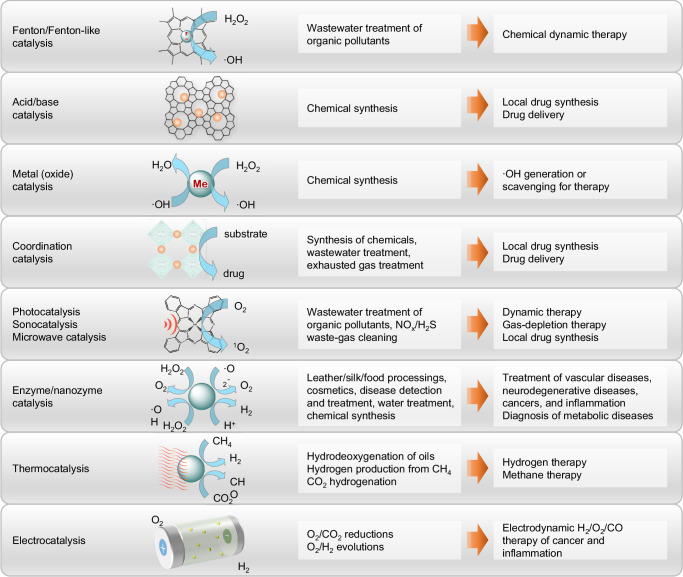
Typical catalytic reactions and their industrial applications for inspiring potential biomedical applications.

Catalysis lies at the heart of many chemical reactions from laboratory to chemical industry. With the development of biomedical field in last decades, many catalytic reactions originating from chemical industry have shown great promise for biomedical applications, and meanwhile pose the specific requirements to biomedical catalysts. Based on the type of catalysts, catalytic reactions can be generally classified as Fenton/Fenton-like catalysis, acid/base catalysis, metal oxide catalysis, transition metal catalysis, coordination catalysis, enzyme/enzyme-mimic catalysis, and photo/ultrasound/electrical/microwave/thermal catalysis. As demonstrated in Fig. 1 and Supplementary Table 1, we summarize the typical catalytic reactions and corresponding catalysts applied in industry and (potentially) in medicine in this section in order for inspiring learning from industrial catalysis.

Fenton/Fenton-like catalysis has flourished in the field of environmental protection, for example, wastewater purification and the remediation of contaminated soils (Supplementary Table 1)^6^. The catalytic mechanism involves a cascade of reactions initiated by the degradation of hydrogen peroxide (H_2_O_2_) in the presence of catalyst such as Fe^2+^ and Cu^2+^ to produce abundant hydroxyl radical (∙OH), which has a stronger oxidation capability than H_2_O_2_. The amplification of oxidation achieves higher efficacy of organics degradation, which is highly dependent on pH value, H_2_O_2_ concentration, temperature, and catalyst dosage. It is well known that both H_2_O_2_ and lactic acid are over-expressed in tumor, providing primary requirements to apply Fenton/Fenton-like catalysis for cancer therapy. Therefore, Fenton/Fenton-like catalysts such as Fe nanoparticles (NPs) have been applied to selectively kill tumor *via* intratumoral delivery (Fig. 1)^1,7^. In addition, some physical fields such as light, ultrasound and microwave can assist Fenton/Fenton-like reactions for enhanced cancer therapy^8^.

Acid/base catalysis plays an important role in chemical industry. Industrially, H_2_SO_4_ is used for the catalytic hydrolysis of cellulose into fermentable sugars for bioethanol production, and NaOH can catalyze the transesterification of vegetable oils with methanol in the biodiesel process (Supplementary Table 1). Zeolites, as porous solid acid catalysts, are widely used in crude oil processing and petrochemistry such as cracking, isomerization, oligomerization, and alkylation under harsh conditions due to their high thermal stability and plentiful surface-active sites^9^. It implies that zeolite NPs can serve as both drug carrier and therapeutic catalyst (Fig. 1). Acid/base catalysts have exceptional ability of donating or attracting electron/proton, and therefore polyoxometalates (POMs) containing multivalent transition metal ions have acid-responsive assembly behavior and enzyme-like catalytic activity, exhibiting a potential biomedical value in the fields of anti-bacteria, anti-inflammation and anti-cancer. Furthermore, chiral acid/base catalysts will enable the catalytic synthesis of chiral drugs in the body for potential therapy.

Both transition metal (e.g. Fe, Ni, Pd, Pt, Mn, Rh, Ru, and Mo) and transition metal oxide (e.g. Fe_2_O_3_, MoO_3_, Bi_2_O_3_, Cr_2_O_3_, and V_2_O_5_) catalysts are mainly used to catalyze redox reactions in environmental and chemical industry. Typically, the hydrogenation of benzene to cyclohexane and the oxidation of benzene to maleic anhydride are catalyzed by Ni and V_2_O_5_/MoO_2_/Al_2_O_3_, respectively (Supplementary Table 1). Importantly, the catalytic activities of transition metals and metal oxides are significantly enhanced when their particle size is decreased to nanoscale due to the increase of specific surface area and surface/inner defects, facilitating the exposure and enhancement of active sites for catalytic reactions, where single-atom catalysis is a typical representation. In fact, many of them have been developed for functional therapeutic agents such Fe and Pd NPs for tumor-targeted hydrogen delivery (Fig. 1)^10–13^. Catalytic reactions based on them for therapy is worth exploring and utilizing.

Coordination catalysis is also widely involved in the industrial field^14^. Coordination catalysts generally are transition metal complexes formed through coordination chemistry. Industrially, [RhCl(PPh_3_)_3_] and [HRh(CO)(PPh_3_)_3_] are intensively applied for catalytic hydrogenation, and [RhI_2_(CO)_2_]^−^ is often employed to catalyze the carbonylation of methanol to acetic acid (Supplementary Table 1). Such hydrogenase-like coordination catalysts can possibly be utilized for catalytic hydrogen production for hydrogen therapy of inflammation-related diseases. Metal-organic frameworks (MOFs), as typical coordination polymers, have been recognized as potential catalysts with high selectivity for CO_2_ fixation and transformation, such as the cycloaddition, reduction and carboxylation of epoxides^15^. MOFs have been intensively developed for drug delivery owing to high specific surface area and flexible biodegradation, and their applications in catalytic medicine such as local drug synthesis can also be possibly explored (Fig. 1).

Enzymes, known as protein biocatalysts, are generally much more efficient than chemical catalysts and usually exhibit high selectivity. However, among approximate 4000 known enzymes, only about 40 of them are actually used for industrial processes because harsh industrial conditions (such as high temperature, high pressure, high acidity/alkalinity and the presence of organic solvents) will quickly inactivate them. Typically, amylase is widely used for the hydrolysis of starch in industry and the prediction of inflammation diseases (pancreas, diabetes, perforated peptic ulcer, *etc.*) in the biomedical field (Fig. 1 and Supplementary Table 1)^16^. Natural enzymes possess intrinsic advantages in biocompatibility for biomedical applications, but are unstable and subject to being degraded and inactivated. Some enzymes including glucose oxidase (GO_x_), horseradish peroxidase and catalase have been encapsulated into nanocarriers for protected delivery for biomedical applications^17–19^. But lots of enzymes are still unrecognized in functions and undeveloped for applications.

As an emerging branch of nanocatalytic medicine^20^, nanomaterial-based artificial enzymes (nanozymes) are designed to mimic the catalytic function of natural enzymes in recent years, providing an alternative strategy to overcome the limitation of natural enzymes^21^. Such nanozymes have similar catalytic properties to natural enzymes, but show much higher stability in great favor of their various applications both in industry and in medicine. Fe_3_O_4_, Au and polypyrrole NPs and graphene oxide (GO) nanosheets possess a unique peroxidase-like property, while Pt, Pd, CeO_2_ and MnO_2_ NPs have inherent catalase (CAT)-/superoxide dismutase (SOD)-mimic ability (Supplementary Table 1). By integrating the catalytic property with other inherent physicochemical nature together, nanozymes are opening a new horizon for their industrial and biomedical applications^20,21^. As an emerging field, the discovery and development of new nanozymes to mimic the versatility of various natural enzymes are in their early stage and attract increasing attention. Noticeably, nanocatalytic medicine covers the content of nanozymes, providing inspirations for engineering nanozymes.

While some catalytic reactions can take place spontaneously under physiological conditions, some reactions require additional energy input to initiate and accelerate the reaction process. Light, ultrasound (US), electricity, magnetism, microwave (MW) and heat are typical stimuli to trigger catalytic reactions^3–5^. Photocatalysts harvest extrinsic optical energy such as solar energy to initiate catalytic reactions for industrial and environmental applications including water splitting hydrogen generation, pollutant degradation, and CO_2_ reduction, etc^22^. Many semiconductor nanomaterials such as TiO_2_, ZnO, SrTiO_3_, CeO_2_, WO_3_, Fe_2_O_3_, GaN, Bi_2_S_3_, CdS and ZnS can act as photocatalysts due to their unique energy bands (Supplementary Table 1)^23^. As a typical application example of photocatalysis in the biomedical field, photodynamic therapy (PDT) exploits cytotoxic ROS to damage cellular lipids, proteins and DNA, and consequently to cause the apoptosis of tumor cells (Fig. 1)^24^. Similar to light, US can also trigger some catalytic reactions. Sonodynamic therapy (SDT) has been an alternative therapeutic strategy to overcome the tissue-penetration limitation of PDT^25^. It is worth noting that most of photosensitizers (e.g. TiO_2_, rose Bengal derivative, indocyanine green, and porphyrin) are potential sonosensitizers. Microwave and electricity can also be employed as stimulus to assist some catalytic reactions^26,27^. The thermal effect of microwaves can produce a large number of hot spots to accelerate catalytic reactions, while the non-thermal effect caused by the microwave−catalyst coupling effect can generate hydroxyl radicals (∙OH) with high oxidation potential. Thermocatalytic decomposition of methane has been one of the most economical hydrogen production methods. Electrocatalysis is used to facilitate the charge transfer between electrode and reactant in electrochemical reactions^28^. Electrochemical therapy (EChT) has been used clinically for cancer therapy by low-voltage direct current to destruct solid tumors.

## Distinguishing: similarities and differences between industrial catalysis and catalytic medicine

Catalytic medicine and industrial catalysis have similarities in catalytic mechanism and methodology, but we have to distinguish differences between them in executive aim and conditions (e.g. pH, solvent, temperature, pressure, *etc.*) (Table 1). The catalytic reactions in industry mainly pursue production efficacy and quality by frequently creating severe reaction conditions such as organic solvent, high temperature, high pressure, strong acidity/basicity, and high energy input. By contrast, the catalytic reactions in the biological environment generally occur under relatively mild conditions (e.g. physiological saline, physiological/pathological pH, body temperature, atmospheric pressure, and low energy input) in order to promise high biosafety. Even so, it is still worthy to compromise the efficacy as the effective dose of generating or consuming therapeutic/diagnostic agents (nM level) is far lower than desired industrial yield (kg scale). Moreover, the catalyst exposure conditions for industrial and medical applications are much different. Many of eco-friendly industrial catalysts have unexpected high toxicity if used for disease treatment. The recycling of catalysts is frequently considered in industry, while for medical applications, the in vivo biodistribution, degradation and metabolism behaviors of catalysts need more attention based on the consideration about their biosafety. In this section, we distinguish and summarize the similarities and differences in catalytic reactions for industrial and medical applications (Table 1) in favor of critical learning from industrial catalysis, deep understanding of catalytic medicine, and rational designing of appropriate catalysts and catalytic reactions for various medical applications.

**Table 1:** Comparison of catalytic reaction conditions for industrial and medical applications

Reaction type	Industrial application condition	R^a^	Medical application condition
Fenton/Fenton-like catalysis	Optimal pH 2.8 − 3.5 Generally RT The right ratio of H_2_O_2_:Fe^2+^ UV light/US/MW assistance	≠ ≠ < >	Stomach pH 1.2; tumor pH 6.8 − 7.4; intestinal pH 7.4 − 9; wound pH 5 − 8 37 °C Tunable ratio of H_2_O_2_:Fe^2+^ Biosafe light/US/MW assistance
Acid/base catalysis	Homogeneous/heterogeneous 25 − 600 °C 1 − 3 atm From strong acid to strong base	> > > >	Heterogeneous 37 °C 1 atm Weak acid and base
Transition metal/metal oxide catalysis	200 − 600 °C 1 − 500 atm Eco-friendly	≠ > <	37 °C 1 atm Bio-friendly
Coordination catalysis	70 − 200 °C 2 − 60 atm	≠ ≠	37 °C 1 atm
(Mimic) Enzyme catalysis	0 − 50 °C Various pH Simplified aqueous solution	> ≈ ≈	37 °C Various pH Physiological/pathological environment
Photocatalysis	Full spectrum of solar light, UV light > 0.1 W/cm^2^	≠ ≠	NIR light, X-ray, self-luminescence <2 W/cm^2^
Sonocatalysis	20 − 200 kHz <110 dB	≠	Diagnosis: 0.8 − 20 MHz, <50 J/cm^2^, <0.1 W/cm^2^; therapy: 0.1 − 3 MHz, 0.125 − 3 W/cm^2^ LIFU, 5 − 1000 W/cm^2^ HIFU
Electrocatalysis	0 − 60 °C, AC 220/380 V	≠	37 °C, DC < 10 V
Microwave catalysis	915 MHz, 2.45 GHz (<1.5 kW) Transiently <5 mW/cm^2^	≠	433 MHz, 915 MHz (<500 W), 2.45 GHz (<200 W), 36.5 − 100 GHz (<120 mW)
Thermocatalysis	350 − 950 °C, 8 − 30 MPa	≠	<42 °C, 1 atm

^a^The symbols “≈”, “≠” and “<”/“>” represent three kinds of relationship between industrial application conditions and medical application conditions: “similar,” “totally different,” and “inclusive,” respectively.

Fenton/Fenton-like catalysis can work in a wide range of pH values, but considering the quantum yield of ∙OH, the optimal pH condition is around pH 3. For industrial wastewater treatment, pH 2.8 − 3.5 is often adopted in order to achieve the highest degradation efficiency^6^. Fortunately, under various physiological/pathological pH conditions, the efficiency of Fenton/Fenton-like catalysis is high enough for disease treatment such as specific killing of tumor cells owing to high cytotoxicity of ∙OH^8^. Because H_2_O_2_ undergoes thermal decomposition into O_2_ at above 50 °C, Fenton/Fenton-like reaction is often carried out at mild temperatures such as room temperature (RT) in industry, and therefore can also well work at body temperature for medical applications. Regarding H_2_O_2_ consumption, a large amount of H_2_O_2_ is needed to achieve a satisfactory yield in industrial applications. Since the in vivo level of H_2_O_2_ is limited, some prodrugs responsive to tumor acidic microenvironment such as MnO_2_ and CaO_2_ can be delivered with Fenton/Fenton-like nanocatalysts together. Even transitional metal carbonyls can play dual roles as both a Fenton-like catalyst and a ∙OH-responsive prodrug of therapeutic CO gas. In order to boost the efficiency of Fenton/Fenton-like catalysis, light/US/MW can be used to assist catalytic reactions and their parameters involving wavelength, frequency and power density have to set to be biosafe^8^. Typically, high energy of ultraviolet (UV) light and full-spectrum solar light are favorable for industrial application, but medical application prefers low intensity of near infrared (NIR) light in order to obtain high tissue penetration capability and avoid phototoxicity.

The distinction of these key parameters is also applicable in photocatalysis, sonocatalysis, electrocatalysis, microwave catalysis and thermocatalysis. For photocatalysis, high NIR-catalytic activity of photocatalysts is highly desired by both industrial and medical applications because of high energy proportion of NIR light in the solar spectrum (>50%) and high biosafety of NIR light. In order to achieve higher tissue penetration depth, X-ray and self-luminescence also have been exploited. Typically, such nanocatalysts are sensitive to X-ray or assisted by chemiluminescence/bioluminescence and scintillating nanoparticles (SCNPs) which can convert X-ray to UV–visible light^29,30^. In consideration of biosafety rather than industrial eco-friendliness, many parameters including frequency, power density, temperature and pressure for medical applications of photocatalysis, sonocatalysis, electrocatalysis, microwave catalysis and thermocatalysis are much different from that for industrial applications (Table 1).

High temperature and pressure are frequently used to enhance the catalytic efficiencies of acid/base catalysis, transition metal/metal oxide catalysis and coordination catalysis in industry. However, in the context of medical applications, body temperature and standard atmospheric pressure cannot achieve the industrially catalytic efficiencies. Therefore, the enhancement of catalyst performance in the physiological conditions and the selection of suitable catalytic routes are vitally important and challenging at present, which needs broader and deeper investigation. In addition, some catalytic reactions which seem low efficiency and no use in industry might be worthy to medical applications because human body is highly sensitive to slight change of in vivo microenvironment. Typically, free radical initiated polymerization can be amplified for controlled cancer treatment in vivo^31^.

Inspired from biology, enzymes are collected and utilized in industry by creating the similar reaction conditions for efficient production of various chemicals, and enzyme-mimic nanocatalysts (nanozymes) are also developed to enhance the stability and multifunctionality of catalysts. In turn, these enzymes and nanozymes can also be facilely used for medical catalysis with the similar reaction conditions^20,21^. Even so, there are still large amounts of enzymes needing to be developed for both industrial and medical applications. Moreover, to engineer unique nanozymes and uncover the enzyme-like function and multimodal activity of nanocatalysts also attract broad attention in the biomedical field.

## Application: translation from industrial catalysis to nanocatalytic medicine

**Fig. 2:**
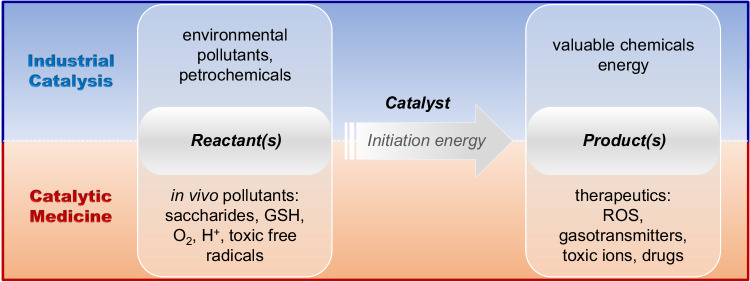
The strategy of catalytic therapy by the translation from industrial catalysis to nanocatalytic medicine.

Learning the principle and characteristic of industrially catalytic reactions and distinguishing the similarities and differences in catalytic reactions for industrial and medical applications are helping us to rationally design appropriate catalysts and catalytic reactions for various biomedical applications. In this section, we summarize the translation from industrial catalysis to nanocatalytic medicine. We have recognized that a catalytic reaction involves three key parameters: reactant(s), product(s) and catalytic initiation energy, and both the generation of therapeutic products and the consumption of specific substrates (in vivo pollutants) can be designed to result in therapeutic effects (Fig. 2). Therefore, in this section, we categorize and summarize catalytic therapy into two strategies, (1) catalytic generation of therapeutic products including ROS, therapeutic gas molecules, toxic ions, and synthetic drugs (Section 4.1), and (2) catalytic consumption of endogenous substrates including saccharides, O_2_, reductive glutathione (GSH), H^+^, and toxic free radicals (Section 4.2). In addition, a variety of endogenous and exogenous stimuli as catalytic initiation energy were also discussed based on the importance of catalytic control over therapy outcome.

### Catalytic generation of useful products: from chemicals to ROS, gasotransmitters, toxic ions and drugs

**Fig. 3:**
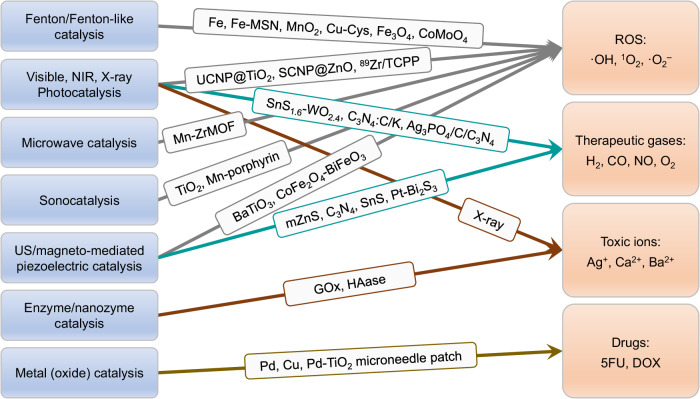
The developed catalytic reactions and catalysts for catalytic generation of therapeutic products.

ROS including ·O^2–^, ·OH, RO·, ROO·, H_2_O_2_ and ^1^O_2_ play an important role in redox balance maintenance, and any aberrance of redox balance in cells may lead to a pathological process, such as oxidative stress-induced apoptosis, which is used to kill cancer cells and bacteria^5^. Typically, photocatalysis is widely utilized to degrade organic contaminants under solar energy in industry and to oxidize organelles by photocatalytically generated ROS under irradiation of visible/NIR light (such as PDT) in medicine. In order to efficiently utilize the solar energy, enhance tissue penetration and lower phototoxicity, the development of nanocatalysts with high NIR-catalytic activity is pursued but challenging at present. On the other way, the upconversional, scintillating and Cerenkov strategies have been used to locally convert NIR light and X-ray into UV lights for UV-sensitive photocatalysts by integration of UCNP (upconversion nanoparticle), SCNP (scintillating nanoparticle) and radionuclide with UV-sensitive photocatalysts (TiO_2_, ZnO or TCPP) (Fig. 3)^32–35^ In addition, local synthesis of RNS from ROS and NO can further amplify the efficiency of cancer therapy because RNS had higher oxidation^36^. In addition to NIR, other excitation sources with stronger tissue penetration such as US, MW and magnetic field have also been applied for catalytically controlled ROS generation. Many photosensitizers such as TiO_2_ and Mn-porphyrin were developed as sonosensitizers for sonodynamic therapy (SDT) based on US-medicated sonocatalysis similar to photocatalysis (Fig. 3)^37,38^. Interestingly, Meng et al. recognized Mn-doped zirconium metal–organic framework (Mn-ZrMOF) nanocubes as a microwave sensitizer enabling microwave dynamic therapy (MDT)^39^, and also co-loaded ionic liquid (IL) as an MW-heating sensitizer and Ga_3_In liquid metal (LM) supernanoparticles as a microwave sensitizer into mesoporous ZrO_2_ NPs for combination of microwave thermal therapy (MTT) with MDT (Fig. 3)^40^. Recently, Shi *et al*. developed US/magnetostriction-mediated piezoelectric nanocatalysis based on BaTiO_3_ and CoFe_2_O_4_–BiFeO_3_ NPs to catalytically generate ROS for cancer therapy, presenting a new strategy of US/magnet-controlled nanocatalytic medicine (Fig. 3)^41,42^.

Fenton/Fenton-like catalysis that has been successfully applied in municipal wastewater treatment can be introduced in catalytic medicine, owing to high oxidizability of catalytically generated ·OH^6^. Instead of direct use of free metal ions in industry, metal-based NPs favor the tumor-targeted delivery of metal to induce the ferroptosis, cuproptosis and zincosis of cancer cells by Fenton/Fenton-like catalysis^43–48^. By utilization of acidic microenvironment in solid tumor, Shi et al. developed acid-responsive amorphous iron nanoparticles and Fe-incorporated mesoporous silica nanoparticles (MSN) as Fenton catalyst precursors for chemodynamic therapy (CDT) of cancer^1,44^. Similarly, Fenton-like nanocatalysts such as MnO_2_, Fe_3_O_4_ NPs and CoMoO_4_ nanosheets can be delivered into tumor for CDT by Fenton-like reaction (Fig. 3)^45–48^. Such a local Fenton catalysis strategy based on nanocatalysts provides a candidate for highly specific cancer therapy. Generally, the therapeutic strategy of catalytic ROS generation draws inspiration from the well-established practices of industrial catalysis, becoming a new-generation therapeutic method.

Some endogenous gasotransmitters including NO, CO, H_2_S, O_2_ and H_2_ are vital in many physiological and pathological processes, exhibiting wide-spectrum anti-inflammation and anti-cancer effects^49^. These gas molecules are generally generated under catalysis of enzymes such as iNOS, HO1 and CBS^50^. The delivery of their substrates is expected to enhance catalytic generation of them in the body, but commonly lack focus-targeting capability. Therefore, the development of artificial nanocatalysts for site-specific stimuli-controlled catalytic generation of therapeutic gas molecules attracts increasing attention. Photo-/sono-/electro-/WM-catalysis are frequently used for wastewater treatment but rarely for production of gases expect H_2_ in industry because of low efficiency of catalytic gas production. Zhang *et al*. constructed the Z-scheme nanocatalysts based on Ag_3_PO_4_/carbon dots-decorated C_3_N_4_ (Ag_3_PO_4_/C/C_3_N_4_) to realize the photocatalytic reduction of endogenous CO_2_ and NO_3_^-^ into CO and NO, respectively, under irradiation of 660 nm laser for cancer therapy (Fig. 3)^51,52^. In order to enhance the tissue penetration capability, we recently developed a Z-scheme SnS_1.68_-WO_2.41_ nanocatalyst, a C/K-incorporated carbon nitride sheet (C_3_N_4_:C/K) and a hydrogen-incorporated titania (TiO_2_:H) nanorod to achieve NIR-photocatalytic generation of H_2_ for hydrogen therapy (Fig. 3)^53–56^. Compared to photocatalysis, sono-/magneto-/WM-catalysis are more desired for catalytic gas generation because US, WM and magnetic fields have higher tissue penetration capability compared with light. Piezoelectric nanocatalysis has been developed to enhance the efficiency of chemical reactions recently^57–59^. In industry, pressure for piezoelectric nanocatalysis is generally offered by ball milling^60^. Inspired from the characteristics of pressure-initiated diseases such as pressure ulcer, we think that piezoelectric nanocatalyst can be used for local piezocatalytic generation of therapeutic gas molecules for their treatment. In addition to the localized pressure, the cavitation effect of US can also cause instantaneous but intense pressure to mediate piezocatalytic generation of gas with mesocrystalline ZnS nanoparticles, C_3_N_4_ nanosheets, SnS nanosheets and Pt-Bi_2_S_3_ heterostructure (Fig. 3)^61–65^. High biosafety and high sonopiezocatalytic efficiency as two key factors have to be carefully considered for biomedical applications. Moreover, magnetostriction-mediated piezoelectric and thermoelectric nanocatalysis based on magnetostrictive-piezoelectric/thermoelectric nanocatalysts should be greatly valuable but have not been reported for piezocatalytic generation of therapeutic gas molecules.

Many transition metal ions have shown a potential for disease treatment by disturbing intracellular redox homeostasis. For instance, Pt^II^, As^III^, Co^III^ and Au^I^ ions are able to strongly bind with sulfur-/seleno-amino acids and DNA, leading to significant inhibition of DNA/protein replication and transcription^66–68^. Typically, Fe^2+^ induces Fenton-catalytically oxidative damage to cells, causing the ferroptosis which is an emerging type of programmed cell dead different from apoptosis and necrosis^69,70^. Similarly, Cu^+^, Co^2+^, Ba^2+^ and Mn^2+^ can also induce ferroptosis based on their Fenton-like characteristics (Fig. 3)^71^. It could be learned that catalytic route can be used to amplify the cytotoxicity of metal ions. In addition, endogenous metal ions such as Na^+^, K^+^, Ca^2+^ and Mg^2+^ are critical to cell functions, including transport of amino acids, maintenance of intracellular pH, and control of cell volume. Local imbalance of their concentration will cause cellular disorder and even death, which provides an innovative strategy for targeted therapy^72–75^.

The off-target effect of drug delivery commonly occurs, even as for targeted nanomedicines developed at present, leading to severe toxic side effects of therapeutics and limited therapy efficacy. In recent years, the strategy for the local catalytic synthesis of active drugs from low-toxicity precursors becomes an emerging and promising solution to address the issue. This strategy has been used in the industrial production of drugs, where neither the toxicity of precursors nor the biocompatibility of reaction conditions needs to be considered. But the catalytic strategy and approaches are still worthy to be learned for catalytic medicine even if catalytic efficiency might be sacrificed to a certain extent in the physiological/pathological conditions. Site-specific/targeted trigger of catalytic reaction is vitally important, and its specificity depends on the precision of external stimuli and the selectivity of internal stimuli. A wide range of industrially used metal-based nanocatalysts, such as Pd, Au, Cu and Ru nanoparticles, and enzymes (GO_x_), horseradish peroxidase, β-galactosidase) have been used to biorthogonally synthesize a variety of drugs for local disease treatment (Fig. 3)^14,76–79^. It has to be noted that the off-target effect of nanocatalysts is unavoidable according to present technological level. In order to avoid the undesired toxicity of nanocatalyst and target-off drug, Gu *et al*. constructed a bioorthogonal catalytic patch comprising a microneedle array patch integrated with Pd nanoparticles-conjugated TiO_2_ nanosheets, which can locally activate a systemically administered prodrug, *N*-allyloxycarbonyl-caged doxorubicin (alloc-DOX), into DOX for melanoma treatment (Fig. 3)^80^. Importantly, this device was robust and removable, and thus completely avoided the in vivo residual of nanocatalysts and consequent toxicity risk. It can be envisioned that bioorthogonal catalytic patch could be a promising tool for high-efficacy and low-toxicity treatment of superficial diseases.

### Catalytic clearance of harmful substrates: from organic pollutants to saccharides, O_2_, GSH, H^+^ and toxic free radicals

In addition to traditionally prescribing medicine according to the disease, the partial or complete removal of some disease-related endogenous substances in vivo, especially excessive toxic species such as toxic radicals, balance-maintaining chemicals such as GSH and H^+^/OH^-^, and necessary fuels such as glucose and oxygen, can yield unexpected outcomes of treatment. Therefore, catalytic consumption of disease-related endogenous substrates in diseased microenvironment based on special catalyst presents a strategy for disease treatment. This section summarized this strategy according to three types of disease-related endogenous substrates.

**Fig. 4:**
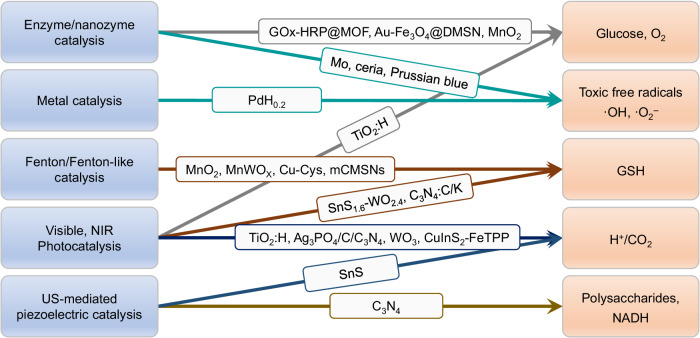
The developed catalytic reactions and catalysts for clearance of harmful substrates.

Glucose and O_2_ are two of most important energy sources to support body metabolism. The GO_x_-catalyzed oxidation of glucose is one of commonest routes to initiating energy generation. Catalytic routes to quick exhaustion of these fuels in tumor will greatly favor tumor starving therapy. Tumor-targeted delivery of GO_x_ by porous carriers including MSN and MOF was proposed to locally exhaust glucose and oxygen in tumor for tumor starving therapy^17,81^. In addition, some GO_x_-mimic nanocatalysts such as MnO_2_ nanosheets have been developed for tumor-targeted catalytic consumption of glucose and oxygen (Fig. 4)^81–83^ Moreover, other major energy sources of cancer cells such as lactic acid (LA) are also envisioned to be able to be deprived by catalytic oxidation through designing the nanocatalyst with a high oxidation potential.

The body is a dynamic and homeostatic system, mainly involving redox homeostasis and acid–base homeostasis, whose persistent imbalance will initiate abnormal reaction and even diseases. GSH as one of major modulators of cellular anti-oxidation defense system (ADS) plays an important role in maintaining cellular redox balance for dynamic repair of cellular injury from oxidative stress, while powerful acid/base-buffering ability of body liquid ensures acid–base homeostasis in various organs to maintaining normal physiology and cell metabolism. Many diseases such as tumor, inflammation and biofilm are generally accompanied with local metabolic disorder such as redox and acid–base imbalances^84–87^. Therefore, local consumption of excessive GSH and H^+^ in the tumor microenvironment can cause oxidative stress to induce tumor apoptosis and enhance therapeutic sensitivity of tumor, becoming an emerging strategy of cancer treatment^88,89^. Traditional direct administration of balance-modulating drugs cannot ensure completely-targeted delivery of drug and thus would bring side effects to normal organs. Fortunately, it has been recognized that catalytically generated holes and ROS on the surface of nanocatalysts are able to oxidize GSH into oxidative glutathione (GSSG) when valence band of nanocatalysts is enough low (≥0.32 eV for GSH-to-GSSG evolution vs normal hydrogen electrode (NHE)), while catalytically separated electrons with enough high reductive capability (≥0 eV for H^+^-to-H_2_ evolution, ≥0.53 eV for CO_2_-to-CO evolution vs NHE) can be used to in situ reduce endogenous H^+^/CO_2_ into H_2_/CO (Fig. 4)^51,53–56,90,91^. In addition to direct consumption of H^+^, depletion of CO_2_ from the HCO_3_^-^/CO_2_ buffer system of body can also destroy acid–base balance. Therefore, catalytic consumption of GSH, H^+^ and CO_2_ becomes an efficient and safe strategy for therapy. Notably, the catalytic reduction of CO_2_ into fuels and chemicals is significant to energy source and environment, and attracts intensive attention. The related catalytic routes provide inspiration to develop suitable catalysts for biomedical applications. In addition to direct oxidation of GSH by holes, ∙OH from Fenton/Fenton-like catalytic reaction can also oxidize GSH into GSSG^92,93^.

Free radicals have essential biological functions such as signal transduction and chemical modification, but excessive amounts of free radicals, especially highly toxic ones such as ·OH and ·ONOO^-^, can cause oxidative stress (OS) and induce a number of OS-related diseases such as neurodegenerative diseases and inflammation. Therefore, the scavenging of excessive toxic free radicals is a well-known therapeutic strategy of protecting normal cells/tissues from oxidative damage. Among various approaches, catalytic scavenging of excessive toxic free radicals holds obvious advantages in scavenging efficiency, sustainability and selectivity, and thus is being developed as an emerging strategy for disease treatment. Noble metal nanocrystals, such as Pt and Pd, and their nano-alloys have been used extensively as hydrogenation catalyst in industry. Based on their unique catalytic hydrogenation characteristics, we synthesized a kind of small-sized Pd hydride (PdH_0.2_) nanocubes at RT and under atmospheric pressure, and used them as ·OH scavenger to eliminate Alzheimer’s disease (AD) inflammation, efficiently blocking synaptic and neuronal apoptosis and ameliorating the cognitive impairment in AD mice (Fig. 4)^94^. In addition to noble metal nanocrystals, some mixed-valence-state rare earth metal oxide nanoparticles such as ceria, Mo and Prussian blue have enzyme-like activity and thus also become an alternative candidate as free radical scavengers (Fig. 4)^95–100^. The strategies for catalytic consumption of toxic free radicals should be valuable for many OS/inflammation-related diseases.

It is worth noting that the band structure of nanocatalyst can be tailored to obtain right potentials of catalytic redox which enable both the consumption of harmful substrates and the generation of therapeutic products^53–56,61–65^. Some harmful substrates such as GSH and LA in the tumor and biofilm microenvironments, LA in the arthritic synovial microenvironment, glucose in the infected diabetic wound, and polysaccharides and NADH in the biofilm can be utilized as sacrificial agents of hole to be locally oxidized/consumed in support of catalytic generation of H_2_. Such substrate depletion can regulate the microenvironments of tumor, biofilm, arthritic synovium and diabetic would, while H_2_ generation can inhibit the respiration of tumor cells and bacteria, promote the proliferation and migration of skin cells, and repolarize related macrophages, realizing an amazing synergistical therapy. Besides H_2_ evolution, conduction band can also be designed to generate other therapeutic agents such as CO and NO. More importantly, this is a “a-stone-two-birds” platform strategy able to address both the symptoms and root cause of complexed diseases. The exploration of nanocatalysts with both a high biosafety and a high therapeutic validity is the key.

## Outlook

Catalysts as an emerging type of activable therapeutic agents are much different from traditional drugs, and catalytic therapy has demonstrated unique advantages in selectivity, efficiency, sustainability and targetability. The increasing demand on catalytic nanomedicines is stimulating interdisciplinary study, presenting an ever-evolving toolbox for innovative methodologies and materials. However, some challenges remain to be addressed to promote the advancement of nanocatalytic medicine.

1. Further deep understanding and broad application of catalytic medicine needs to be continually strengthened. Although we have here summarized general industrial catalysts and catalytic reactions for inspiring learning and also distinguished the similarities and differences between industrial catalysis and medical catalysis, there still is a big gap between understanding and application of catalytic medicine. We urge researchers across diverse disciplines to recognize that some often overlooked low-efficiency catalytic reactions could be of great value to medical applications due to the profound amplification effect of catalysis. It is also essential to reassess/modulate industrial catalysts and catalytic reaction conditions for medical use for ensuring biosafety standards are met. Only a few of medical applications of catalysis have been developed, leaving a big margin worthy to be exploited in the future. Moreover, specific catalyst and catalytic reactions need to be customized to meet the particular demands of a diversity of biomedical applications, including the treatment of various diseases. We have summarized a series of strategies for catalytic therapy in this review, which is expected to provide inspiration for catalytic medicine customization.

2. Some merits of catalytic medicine are worth noting, mainly including no need of any toxic drug, high selectivity, and high precision of catalytic reactions which can be temporospatially controlled accurately by external stimuli and thus avoid potential side effects mediated by catalyst off-target. If endogenous substrates such H_2_O, CO_2_, glucose and GSH are used, high sustainability for catalysis can be realized in great favor of long-term and high-dosage treatment. Besides photocatalysis, sono-/WM-/magneto-catalysis are more intriguing because of higher tissue penetration capability of these stimuli, but related reports are quite a few, which is mainly limited by the difficulty in the engineering of highly sensitive catalysts. Widening the pool of catalytic reactions and building an ever-evolving toolbox of nanocatalysts are urgently necessary, when high-throughput screening technology by integrating microfluidic chip with artificial intelligence (AI) holds a potential for the efficient selection of catalysts. Nonetheless, overcoming the challenges in constructing high-quality datasets for catalytic and biochemical experiments, as well as developing automated methods for extracting characteristic parameters and creating scalable/migratory modeling algorithms tailored to diverse catalytic and biological problems, represents a critical undertaking that necessitates interdisciplinary collaboration and cross-learning exploration. Moreover, the biosafety of catalysts should be seriously considered, especially when nanocatalysts remain long in normal organs. As for nanocatalysts with a relatively high risk of biosafety, the removable patch possibly is an ideal tool for superficial diseases. It is worthy of noting that an investigator-initiated trial about the application of TiO_2_:H-based photocatalytic glucose-depleting and H_2_-generating dressing for diabetic foot ulcer treatment is being executed owing to high biosafety and validity of the dressing^54^. In addition, both the scale-up production and the quality control of medical catalysts have to be addressed in the future.

3. Nanocatalysts have a distinct advantage in catalytic efficiency, but the understanding of their catalytic mechanisms and interactions with the body is far unclear. Surface chemistry plays a dominating role in catalysis, and its effect on catalytic behaviors and mechanisms might be changed by the biological microenvironment, causing a mutual influence between catalytic routes and biological microenvironment. The mutual influence also depends on biological microenvironment or the position of nanocatalyst in cells/tissues, where negative effects are necessarily avoided. Main technical challenges of defining in vivo catalytic routes lie to accurately identifying endogenous reactants and catalytic products in real time, both of which are frequently in a dynamic evolution, in a specific biological microenvironment. We envision that the investigation complexity could be reduced by precisely engineering the band structure of catalysts to restrict endogenous reactant (or sacrificial agent), and the development of various bioprobes for in vivo real-time monitoring reactants and products is desired.

4. Application of catalysis in the biological environment also facilitates the fundamental understanding in chemical biology and the development of innovative biomedical tools. It will build a network for catalytic nanomedicine to couple with other therapeutic modalities. One of the promising approaches is engineering nanocatalytic medicine as immune-regulatory agents, utilizing the chemical functions of nanocatalytic therapy to augment downstream immunotherapeutic effects. Deeper understanding of catalytic mechanism and biochemical basis underlying many diseases would be helpful for engineering catalytic nanomedicines. Overall, the utilization of catalytic nanomedicine to control the progression of disease will be a revolutionary technology.

### Supplementary information

Supplementary Information
