# Supplementary material for: Critical learning from industrial catalysis for nanocatalytic medicine
Source: Nat Commun. 2024 May 8;15:3857. doi: 10.1038/s41467-024-48319-9 (PMC11079063; doi:10.1038/s41467-024-48319-9)
Supplement: Supplementary file 1 — Supplementary Information [file 41467_2024_48319_MOESM1_ESM.pdf]

Supplementary Information

Critical Learning from Industrial Catalysis for Nanocatalytic Medicine

Zhaokui Jin,[1,2,†] Lingdong Jiang,[3,†] Qianjun He[1,*]

[1] *Medical Center on Aging, Ruijin Hospital; Shanghai Key Laboratory of Hydrogen Science & Center of Hydrogen Science, School of Materials Science and Engineering, Shanghai Jiao Tong University, Shanghai 200240, China*

[2] *School of Biomedical Engineering, Guangzhou Medical University, Guangzhou 510182, China*

[3] *College of Pharmacy, Shenzhen Technology University, Shenzhen 518118, China*

† These authors contributed equally to this work.

* Corresponding author. Qianjun He (qjhe@sjtu.edu.cn)

Supplementary Table 1. Typical catalytic reactions and catalysts applied in industrial and biomedical fields.

Catalytic type	Typical reaction	Catalyst	Industrial application	Medical application	Ref.
Fenton/Fenton-like catalysis	$Fe^{2+} + H_2O_2 + H^+ \rightarrow Fe^{3+} + \cdot OH + H_2O$	Fe^{2+}, Fe^{3+}, Fe_3O_4, Fe, Fe-Co, Prussian blue analogues, $CoMoO_4$, Fe/Cu/Co-based MOF (*e.g.* MIL-100(Fe), MIL-88B(Fe), MIL-53(Fe), Fe_3O_4@ MIL-100, Fe–Cr-MIL-101, $CuFe_2O_4$/Cu@C, Fe_3O_4/C/Cu, $Fe_{0.8}Co_{2.2}O_4$, Pd@MIL-100 (Fe)	Wasterwater treatment, degradation of pollutants (*e.g.* acid orange 7, bisphenol A, carbamazepine, clofibric acid, congo red, diphenhydramine, methyl orange, methylene blue, Rhodamine B, phenol, phenytoin)	Cancer therapy, anti-inflammation	[1,2]
Acid/base catalysis	triglycerides + $3CH_3OH \rightarrow$ glycerol +3 fatty acid methyl ester	NaOH, KOH, perovskite ($ZnTiO_3$)	Biodiesel production by transesterification	Synthesis of pharmaceutical compounds	[3]
	cellulose $\rightarrow$ sugar	H_2SO_4	Hydrolysis of cellulose to fermentable sugars for bioethanol production	—	[4]
Transition metal (oxide) catalysis	$CO + CH_3OH \rightarrow HCOOCH_3$	Cu NPs	Production of methyl formate from methanol	—	[5]
	$C_2H_4 \rightarrow C_2H_4O$	Ag NPs	Epoxidation reaction of ethylene	—	[5,6]
	$R\text{-}CH{=}CH_2 + H_2 \rightarrow R\text{-}CH_2\text{-}CH_3$<br>$R\text{-}C_6H_5 + 3H_2 \rightarrow R\text{-}C_6H_{11}$	Pt, Pd, Ru NPs	Hydrogenation of C=C bond and arene	—	[5,7]
	$Benz\text{-}NO_2 + 2H_2 \rightarrow Benz\text{-}NH_2 + 2\cdot OH$	Au, Ag, Pd NPs	Hydrogenation of nitro group	Potential cancer therapy by ·OH generation	[5,8]
	$\cdot OH + 1/2H_2 \rightarrow H_2O$	Pd, Pt, Ru NPs	Hydrogenation of ·OH	Anti-inflammation by scavenging ·OH; cancer therapy by inducing oxidation stress	[5,9]
	$C_6H_5Cl + 1/2H_2 \rightarrow C_6H_6 + HCl$	Pd NPs	Dehalogenation	—	[5,10]
	$RCH_2OH + O_2 \rightarrow RCOOH + H_2O$	Pt, Au, Pd NPs	Oxidation of hydroxyl group	Potential oxidative damage for therapy	[5,11]

	$RCHOHR' + 1/2O_2 \rightarrow RCOR' + H_2O$				
	$Br\text{-}Ph\text{-}COCH_3 + Ph\text{-}B(OH)_2 \rightarrow Ph\text{-}Ph\text{-}COCH_3 + BrB(OH)_2$	Pd NPs	Suzuki C-C coupling	—	[5,12]
	$Ph\text{-}C\equiv CH + Ph\text{-}I \rightarrow Ph\text{-}C\equiv C\text{-}Ph + HI$	Pd NPs, Pd complexes	Sonogashira cross-coupling	—	[5,13]
Metal oxide catalysis	$Ph\text{-}CH_2CH_3 \rightarrow Ph\text{-}CH{=}CH_2 + H_2$	Fe_2O_3, CoO_x	Ethyl benzene dehydrogenation to styrene	Potential hydrogen therapy	[14]
	$CO + 2H_2 \rightarrow CH_3OH$	$Cu–ZnO/Al_2O_3$	Methanol synthesis from H_2/CO mixtures	—	[15]
	n-butane → maleic anhydride	$(VO)_2P_2O_7$	Selective oxidation of butane to maleic anhydride	—	[16]
	$RCHO + 1/2O_2 \rightarrow RCOOH$	$FePO_4$	Oxidation of methacrolein to methacrylic acid	Potential detoxification	[17]
	$C_6H_5CH_3 \rightarrow C_6H_6$	MoO_3/Al_2O_3	Demethylation of toluene to benzene	—	[18]
	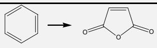	$V_2O_5/MoO_2/Al_2O_3$	Benzene oxidation to maleic anhydride	—	[19]
Coordination catalysis	$RCH{=}CHR' + CO + 1/2H_2 \rightarrow RCH_2CHR'CO$	[Rh(acac)(CO)2]	Hydroformylation of cyclohexene and 2-methylpropene	Forming medical adhesives and sealants	[20]
	$C_6H_5CHO + Me_3SiCN \rightarrow C_6H_5C(CN)(OSiMe_3)$	HKUST-1, $\{[(Cu(L)_2(H_2O)_2](MeOH)_4\}_n$, $[Cu_2(L)(H2O)_2](DMA)_2$, $\{[Zn_2(abtc)(H_2O)_3](DMA)\}_n$, $[Gd_2(bpt)_2(H_2O)_2]\cdot(DMF)_2(H2O)_6$	Catalytic cyanosilylation of benzaldehyde	—	[21]
	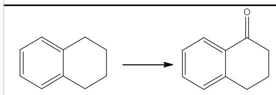	MIL-101(Cr) MIL-101(Fe)	Oxidation of hydrocarbons, wastewater treatment, exhausted gas treatment	—	[21,22]
	$RSR' + 1/2O_2 \rightarrow RSOR'$	MIL-101(Cr)	Oxidation of sulfides	Potential drug synthesis	[21,23]
	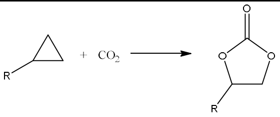	MIL-101(Cr)	Cycloaddition of CO_2 to epoxides, CO_2 conversion	Potential drug synthesis	[21,24]
	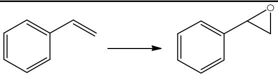	CZJ-4	Epoxidation of styrene	—	[21,25]
Enzyme/nanozyme catalysis	$Fe(II) + H_2O_2 + H^+ \rightarrow Fe(III) + \cdot OH + H_2O$	Haem	Iron supplement, food additive	Chemodynamic therapy	[26]
	protein → peptide	Metalloproteases	Detergent, bio-additive, peptide synthesis, dehairing and destaining	Modulation of tumor cell growth, differentiation, apoptosis, migration and invasion	[27]
	$2\cdot O_2^- + 2H^+ \rightarrow H_2O_2 + O_2$	Superoxide dismutase (SOD); SOD-mimicking nanozymes: Au, Pt, Pd, fullerene, CeO_2, Au/CeO_2, MnO_2, Mn_3O_4, $FePO_4$, molybdenum sulfide, Prussian blue NPs	Food additive, cosmetics	Anti-aging, anti-inflammation, treatment of autoimmune diseases, tumor hypoxia relief	[28]
	$2H_2O_2 \rightarrow 2H_2O + O_2$	Catalase; Catalase-mimicking nanozymes: Au, Ag, Pt, Pd, Ir, BSA-IrO_2, Au@Pt, CeO_2, MnO_2, Mn_3O_4, Fe_3O_4, Fe_2O_3, Co_3O_4, V_6O_{13}, NiPd, Prussian blue NPs	Food packaging, textile, facial care	Tumor hypoxia relief	[28,29]
	$NADPH + 2O_2 \rightarrow NADP^+ + 2\cdot O_2^- + H^+$	NADPH oxidase	—	Treatment of vascular disease, Parkinson's disease, and tumor	[30]
	$GSSG + NADPH + H^+ \rightarrow 2GSH + NADP^+$	GSH reductase	—	Diagnosis of hepatic disease, and tumor therapy	[31]
	$(NH_2)_2CO + H_2O \rightarrow CO_2 + 2NH_3$	Urease	Wastewater treatment	Determination of urea in biological fluids	[32]
	glucose + O_2 → gluconic acid + H_2O_2	Glucose oxidase (GO_x); GO_x-mimicking nanozymes: Cu_2O, Au NPs	Food additive, food freshness preservation	Starvation therapy of tumor, diabetes treatment	[28,33]

	$H_2 \rightarrow 2H^+ + 2e^-$	Hydrogenase	Wastewater treatment, prevention of microbial corrosion	Hydrogen therapy, anti-bacteria, anti-inflammation, Alzheimer's disease treatment	[34]
	$AH + B^+ \rightarrow A^+ + BH$<br>$AH_2 + B \rightarrow A + BH_2$	Dehydrogenase	The conversion of alcohol to aldehyde and acid	Diagnosis of myocardial infarction, liver disease, and certain malignancies	[35]
	$N_2 + 8\ H^+ + 8\ e^- + 16\ ATP \rightarrow 2\ NH_3 + H_2 + 16\ ADP + 16\ Pi$	Nitrogenase	Chemical fertilizer	—	[36]
	$CH_2=CH\text{-}CN + H_2O \rightarrow CH_2=CH\text{-}CONH_2$	Nitrile hydratase	Water addition	—	[37]
	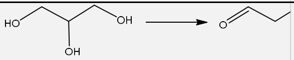	Glycerol dehydratase	Water elimination	—	[38]
	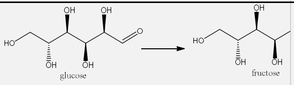	Glucose isomerase	The production of high fructose syrup	—	[38,39]
	cholesterol + $O_2 \rightarrow$ cholest-4-en-3-one + H_2O_2	Cholesterol oxidase	Decrease the cholesterol level in food	Prevention of high lipids, arteriosclerosis, and hypertension	[40]
	lactose + $H_2O \rightarrow$ glucose + galactose	β-Galactosidase	Lactose hydrolysis	Diagnosis of kidney diseases	[40,41]
	polypeptide + $H_2O \rightarrow$ polypeptide fragments	α-Chymotrypsin	Proteolysis	Anti-inflammation, promoting the penetration of antibiotics and chemotherapeutic agents into the lesions	[40,42]
	starch + $H_2O \rightarrow$ maltose	Diastase	Starch hydrolysis	Treatment of indigestion	[40,43]
	uric acid + O_2 + $H_2O \rightarrow$ allantoin + H_2O_2 + CO_2	Uricase	Uric acid biosensor	Treatment of hyperuricemia	[40,44]
	$H_2O_2 + AH_2 \rightarrow 2H_2O + A$	Peroxidase	Rapid deterioration of H_2O_2, water treatment	Scavenging ROS in tumor	[40,45]
	$CO_2 + H_2O \rightarrow H_2CO_3$	carbonic anhydrase	Detection of heavy metal pollution in environment	Diagnosis of colon, kidney, prostate, breast cancers	[46]
	L-asparagine + $H_2O \rightarrow$ L-aspartate + NH_3	Asparaginase	Food additive for the decrease of acrylamide in food	Inhibition tumor growth, treatment of leukaemia	[47]
	Collagen hydrolysis	Collagenase	Cosmetics	Treatment of skin ulcers	[47,48]
	L-Glutamine + $H_2O \rightarrow$ L-glutamate + NH_3	Glutaminase	Food additive	Treatment of leukaemia	[47,49]
	Bacterial cell wall hydrolysis	Lysozyme	Food additive	Antibacterial, antiviral, hemostasis	[47,50]
	RNA hydrolysis	Ribonuclease	—	Antiviral, treatment of arthralgia	[47,51]
	plasminogen $\rightarrow$ plasmin	Streptokinase	—	Blood clots	[47,52]
	protein $\rightarrow$ peptide	Trypsin	Leather manufacturing, silk processing, food processing	Treatment of ulcer, anti-inflammation	[47,53]
	plasminogen $\rightarrow$ plasmin	Urokinase	—	Treatment of blood clots	[47,54]
	binding the rings of benzylpenicillin (penicillin G) and phenoxymethylpenicillin (penicillin V)	Penicillin acylase	—	Antibiotics	[47,55]
	starch $\rightarrow$ sugar	Amylase	Starch processing	Diagnosis of acute inflammation, diabetes	[47,56]
	acetylcholine $\rightarrow$ choline + acetic acid	Acetylcholinesterase	Organophosphorus pesticides and nerve reagent detection	Neuroregeneration	[57]
	triglyceride $\rightarrow$monoglycerides + fatty acids	Lipase	Triolein transesterification, chloramphenicol palmitate synthesis, phenolic acid	Diagnosis of acute pancreatitis	[58]

			ester synthesis		
	lactose → glucose + galactos	Galactosidase	Production of low lactose, milk and galactooligosaccharides	Diagnosis of kidney diseases	[59]
	$A_{red} + O_2 + H_2O \rightarrow A_{ox} + H_2O_2$<br>$A_{red} + O_2 \rightarrow A_{ox} + H_2O$<br>$A_{red} + O_2 \rightarrow A_{ox} + \cdot O_2^-$	Au, Ag, Pt, Pd, Ir, Ru, Au@Pt, Au@PdPt, PtCo, PtCo@MnO_2, NiPd, Se, Pt−Se, ZnO, CeO_2, MnO_2, Mn_3O_4, V_6O_{13}, $CoFe_2O_4$, $MnFe_2O_4$, $NiCo_2O_4$ nanopaticles	Oxidase mimicking nanozyme	ROS production for tumor therapy	[28,60]
	$H_2O_2 \rightarrow \cdot OH$<br>$TMB + H_2O_2 \rightarrow oxTMB + H_2O$	Au, Ag, Pt, Pd, Cu, Ir, Ru, Au@Ag, Au@Pt, Au@Pd, Au/CuS, Au/g-C_3N_4, Pd-Ir, Bi/Au, Bi/Pt, PtCu, Pt-MoO_3, $BiFeO_3$, CuO, CuO/Pt, CuS, $Cu(OH)_2$, $CuInS_2$, Fe_2O_3, Fe_3O_4, $FePO_4$, $FeVO_4$, FeS, Fe_3S_4, FeSe, FeTe, V_2O_5, VO_2, V_6O_{13}, CeO_2, CoP, Co_3O_4, $CoFe_2O_4$, MnO_2, MnSe, $MnFe_2O4$, MoS_2, ZnO, $ZnFe_2O_4$, $ZnFe_2O4$-ZnO, porphyrin-ZnS, NiPd, Zn-CuO, $AgVO_3$, WSe_2, PtPd-Fe_3O_4, polypyrrole, silver halides, gold–graphitic carbon nitride, single-walled carbon nanotube, graphene oxide, Cu_2O, Mn_3O_4, prussian blue NPs	Horseradish peroxidase (HRP) mimicking nanozyme	ROS production for tumor therapy	[28,61]
	$2GSH + H_2O_2 \rightarrow GS–SG + 2H_2O$	V_2O_5, Se, Te, Mn_3O_4 NPs	Glutathione peroxidase (GSH-Px) mimicking nanozyme	Tumor therapy	[28,62]
	$2X^- + 2H^+ + H_2O_2 \rightarrow$ “2X” + $2H_2O$	V_2O_5, CeO_{2-x} NPs	Haloperoxidase mimicking nanozyme	Tumor therapy	[28,63]
	$NO_3^- + H_2O + 2e^- \rightarrow NO_2^- + 2OH^-$	CdS-Pt NPs	Nitrate reductase mimicking nanozyme	Tumor diagnosis	[28,64]
	$SO_3^{2-} + H_2O \rightarrow SO_4^{2-} + 2H^+ + 2e$	MoO_3	Sulfite oxidase mimicking nanozyme	Anti-tumor	[28,65]
	$Fe^{2+} + O_2 + H^+ \rightarrow Fe^{3+} + H_2O$	Cu_2O	Cytochrome c oxidase mimicking nanozyme	Anti-tumor	[28,66]
	L-arginine + $H_2O_2 \rightarrow$ HNO	graphene-haemin	Nitric oxide synthase mimicking	NO therapy of tumor	[28,67]
	Ascorbic acid + $O_2 \rightarrow$ ascorbyl radicals	Au@Pt	Ascorbate oxidase mimicking nanozyme	Anti-tumor	[28,68]
	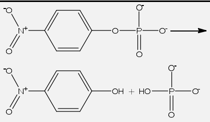	CeO_2	Phosphatase mimicking nanozyme	Diagnosis of liver cancer	[28,69]
Photocatalysis	$PS + hv \rightarrow {}^1PS^* \rightarrow {}^3PS^*$;<br>${}^3PS^* + {}^3O_2 \rightarrow {}^1O_2$;<br>${}^3PS^*$ + substrates → free radicals	Hematoporphyrin (Photofrin®), Protoporphyrin IX (Levulan®), Temoporfin (Foscan®), Verteporfin (Visudyne®), Talaporfin (Laserphyrin®), Chlorin-e6 (Photolon®), Bacteriochlorin, Phthalocyanine, Phenothiazinium salts, Rose Bengal, Squaraines, BODIPY dyes, Phenalenones	Wastewater treatment	Photodynamic therapy of cancer	[70,71]
	Organic Pollutant + $O_2 \rightarrow$ $CO_2 + H_2O$+ Mineral Acid	TiO_2, Fe_2O_3-doped TiO_2, Cu-doped TiO_2, Sn^{2+}-doped TiO_2, Pt/TiO_2, ZnO-graphene, $Zr_xTi_{1-x}O_2$, BiOI/Al_2O_3, Ag/g-C_3N_4, g -C_3N_4/WO_3, Ag-loaded TiO_2, Pd/TiO_2	Photocatalytic oxidation of organic pollutants	Photodynamic therapy of cancer	[72,73]
	$NO + \cdot OH \rightarrow HNO_2$<br>$HNO_2 + \cdot OH \rightarrow NO_2 + H_2O$<br>$NO_2 + \cdot OH \rightarrow HNO_3$<br>$NO + \cdot O_2^- \rightarrow NO_3^-$<br>$3NO_2 + 2OH^- \rightarrow 2NO_3^- + NO +$	Anatase, TiO_2, Fe/TiO_2, Mo-doped TiO_2, Pt-doped TiO_2, Au/CeO_2-TiO_2, g-C_3N_4-$BiVO_4$	Photocatalytic oxidation of NO_x	NO gas therapy of cancer	[72,74, 75]

	H_2O				
	$H_2S + h^+ \rightarrow H_2S^+ \rightarrow HS\cdot + H^+$<br>$\cdot OH + H_2S^+ \rightarrow HS\cdot + H_2O$<br>$HS\cdot + O_2 \rightarrow HSOO\cdot$<br>$HSOO\cdot + O_2 \rightarrow SO_2$	WO_3/TiO_2	Photocatalytic oxidation of H_2S	H_2S gas therapy of cancer	[72,76, 77]
Sonocatalysis	Ground state PS + ultrasound → Singlet excited $^1PS^*$ → Triplet excited $^3PS^*$; Triplet excited $^3PS^* + {}^3O_2 \rightarrow {}^1O_2$; Triplet excited $^3PS^*$ + substrates → Free radicals	Porphyrin-based sensitisers (Protoporphyrin IX, Phthalocyanine, Photofrin, Chlorin e6, ClAl-phthalocyanine), Xanthene-based sensitisers (rose Bengal and its derivative), Acridine orange, Methylene blue, Curcumin, Indocyanine green, IR-780, δ-aminolevulinic acid (ALA), TiO_2, Fe_3O_4, Au@ black phosphorus NPs, Protohemin, Copper–cysteamine	Wastewater treatment	Sonodynamic therapy of cancer	[78,79]
Microwave catalysis	$MW(hv) + catalyst \rightarrow h^+ + e^-$<br>$h^+ + H_2O \rightarrow \cdot OH + H^+$<br>$h^+ + OH^- \rightarrow \cdot OH$<br>$e^- + O_2 \rightarrow \cdot O_2^-$<br>$\cdot O_2^- + H_2O \rightarrow \cdot OOH + OH^-$<br>$2\cdot OOH \rightarrow O_2 + H_2O_2$<br>$e^- + \cdot OOH + H_2O \rightarrow H_2O_2 + OH^-$<br>$e^- + H_2O_2 \rightarrow \cdot OH + OH^-$<br>$\cdot O_2^- + H_2O_2 \rightarrow \cdot OH + OH^- + O_2$	Fe^0, $CoFe_2O_4$, activated carbon powder, Carbon supported copper, Carbon supported platinum, Nickel oxides, Boron doped diamond electrode	Degradation of pentachlorophenol, brilliant green, congo red, p-Nitriphenol, 4-chlorophenol, 2,4-Dichlorophenoxy acetic acid	Microwave dynamic therapy of cancer	[80,81]
Electrocatalysis	$O_2 + 2H_2O + 4e^- \rightarrow 4OH^-$	Graphene, N-doped graphene nanosheets, Porous boron carbon nitride (BCN) nanosheets	Oxygen reduction reaction (ORR)	Electrodynamic therapy of cancer	[82,83]
	$2CO_2 + 4H^+ \rightarrow 2H_2O + 2CO$	MoS_2, WSe_2 nanoflakes,	Carbon dioxide reduction reaction (CO_2RR)	CO therapy of cancer	[82,84]
	$2H_2O + 4e^- \rightarrow 2H_2 + 2OH^-$	Channelled-engineered MoS_2, Double-gyroid MoS_2, Mo_2CT_x,	Hydrogen evolution reaction (HER)	Hydrogen medicine against inflammation and cancer	[82,86, 87]
	$4OH^- \rightarrow O_2 + 2H_2O + 4e^-$<br>(alkaline condition)<br>$2H_2O \rightarrow O_2 + 4H^+ + 4e^-$<br>(acidic condition）	Exfoliated CoCo, NiCo, NiFe, CoFe layered double hydroxides, black phosphorus nanosheets, Te-doped black phosphorus, porous Co_3O_4, NiO stabilized by TiO_2, porous FeNi oxide, γ-CoOOH, CoMn layered double hydroxides, CoNiFe hydroxide, $CoSe_2$, $NiCo_2Se_4$, $Co_{0.4}Fe_{0.6}Se_2$, Co_3S_4, $NixCo_{3-x}S_4$-decorated Ni_3S_2, Ni_3N, Co−Mn carbonate hydroxide, ternary NiCoP, cobalt borate/graphene, NiCo bimetal MOF, NiFe-MOF array, $Ti_3C_2T_x$−CoBDC, N,S-graphene, $g\text{-}C_3N_4/Ti_3C_2$	Oxygen evolution reaction (OER)	Hypoxia relief of tumor and OER enhanced PDT	[82,88, 89]
Thermo-catalysis	$(CH2O)_n + H_2 \rightarrow (CH_2)_n + H_2O$	$Co\text{-}MoS_2/Al_2O_3$, $Ni\text{-}MoS_2/Al_2O_3$, Pd/C, Pd/ZrO_2, $Pt/Al_2O_3/SiO_2$, Pt/ZrO_2 $Rh/ZrO_2Ru/C$, Ru/TiO_2	Hydrodeoxygenation (HDO) of pyrolysis oils	—	[90]
	$(CH_4)_g \rightarrow (CH_3)_a + (H)_a$<br>$(CH_{3-x})_a \rightarrow (CH_{2-x})_a + (H)_a$<br>$2(H)_a \rightarrow (H_2)_g$	Ni, Fe, Ni–Cu, Ni–Cu–Al, Ni/SiO_2, Ni/TiO_2, Ni/Al_2O_3, Ni/La_2O_3, Fe/Al_2O_3, Fe/MgO, $NiCu/Al_2O_3$, $Ni\text{–}Ca/SiO_2$, $Ni\text{–}K/SiO_2$, $Ni\text{–}Ce/SiO_2$	Hydrogen production by thermocatalytic decomposition of methane	Potential hydrogen therapy	[91]
	$CO_2 + 4H_2 \rightarrow CH_4 + 2H_2O$<br>$CO_2 + 3H_2 \rightarrow CH_3OH + H_2O$<br>$CO_2 + 6H_2 \rightarrow CH_3OCH_3 + 3H_2O$	Ni, Co, Fe	CO_2 hydrogenation	Potential methane therapy	[92]

Supplementary References:

[1] Tang, Z., Zhao, P., Wang, H., Liu, Y. & Bu, W. Biomedicine meets Fenton chemistry. *Chem. Rev.* **121**, 1981–2019 (2021).

[2] Cheng, M. et al. Metal-organic frameworks for highly efficient heterogeneous Fenton-like catalysis. *Coord. Chem. Rev.* **368**, 80–92 (2018).

[3] Orege, J. I. et al. Recent advances in heterogeneous catalysis for green biodiesel production by transesterification. *Energy Convers. Manag.* **258**, 115406 (2022).

[4] Hafid, H. S. et al. Over production of fermentable sugar for bioethanol production from carbohydrate-rich Malaysian food waste via sequential acid-enzymatic hydrolysis pretreatment. *Waste Manage.* **67**, 95–105 (2017).

[5] Yan, N., Xiao, C. & Kou, Y. Transition metal nanoparticle catalysis in green solvents. *Coord. Chem. Rev.* **254**, 1179–1218 (2010).

[6] Van Hoof, A. J. F., Filot, I. A. W., Friedrich, H. & Hensen, E. J. M. Reversible restructuring of silver particles during ethylene epoxidation. *ACS Catal.* **8**, 11794–11800 (2018).

[7] Wiesenfeldt, M. P., Nairoukh, Z., Dalton, T. & Glorius, F. Selective arene hydrogenation for direct access to saturated carbo- and heterocycles. *Angew. Chem. Int. Ed.* **58**, 10460–10476 (2019).

[8] Xu, K. et al. Convenient and selective hydrogenation of nitro aromatics with a platinum nanocatalyst under ambient pressure. *Adv. Synth. Catal.* **353**, 1260–1264 (2011).

[9] Tao, G. et al. A strategy of local hydrogen capture and catalytic hydrogenation for enhanced therapy of chronic liver diseases. *Theranostics* **13**, 2455–2470 (2023).

[10] Li, Z. et al. Few-atomic zero-valent palladium ensembles for efficient reductive dehydrogenation and dehalogenation Catalysis. *ACS Nano* **17**, 22859–22871 (2023).

[11] Ide, M. S. & Davis, R. J. The important role of hydroxyl on oxidation catalysis by gold nanoparticles. *Acc. Chem. Res.* **47**, 825–833 (2014).

[12] Díaz-Sánchez, M., Díaz-García, D., Prashar, S. & Gómez-Ruiz, S. Palladium nanoparticles supported on silica, alumina or titania: greener alternatives for Suzuki–Miyaura and other C–C coupling reactions. *Environ. Chem. Lett.* **17**, 1585–1602 (2019).

[13] Platonova, Y. B., Volov, A. N. & Tomilova, L. G. Palladium(II) phthalocyanines efficiently promote phosphine-free Sonogashira cross-coupling reaction at room temperature. *J. Catal.* **391**, 224–228 (2020).

[14] Venugopal, A. K., Venugopalan, A. T., Kaliyappan, P. & Raja, T. Oxidative dehydrogenation of ethyl benzene to styrene over hydrotalcite derived cerium containing mixed metal oxides. *Green Chem.* **15**, 3259 (2013).

[15] Melián-Cabrera, I., Granados, M. L. & Fierro, J. L. G. Pd-modified Cu–Zn catalysts for methanol synthesis from CO_2/H_2 mixtures: Catalytic structures and performance. *J. Catal.* **210**, 285–294 (2002).

[16] Duarte De Farias, A. M. et al. Vanadium phosphorus oxide catalyst modified by Niobium doping for mild oxidation of n-butane to maleic anhydride. *J. Catal.* **208**, 238–246 (2002).

[17] Zheng, Y., Zhang, H., Wang, L., Zhang, S. & Wang, S. Transition metal-doped heteropoly catalysts for the selective oxidation of methacrolein to methacrylic acid. *Front. Chem. Sci. Eng.* **10**, 139–146 (2016).

[18] Kamiguchi, S., Nishida, S., Kurokawa, H., Miura, H. & Chihara, T. Formation of Brønsted acid site on halide clusters of group 5 and 6 transition metals- Catalytic methylation and demethylation of methylbenzenes with methanol. *J. Mol. Catal. A. Chem.* **226**, 1–9 (2005).

[19] Bielański, A. & Najbar, M. V_2O_5-MoO_3 catalysts for benzene oxidation. *Appl Catal A-GEN.* **157**, 223–261 (1997).

[20]Weber, L. Phosphorus heterocycles: From laboratory curiosities to ligands in highly efficient catalysts. *Angew. Chem. Int. Ed.* **41**, 563–572 (2002).

[21] Wu, C. & Zhao, M. Incorporation of molecular catalysts in metal–organic frameworks for highly efficient heterogeneous catalysis. *Adv. Mater.* **29**, 1605446 (2017).

[22] Santiago-Portillo, A. et al. MIL-101 as reusable solid catalyst for autoxidation of benzylic hydrocarbons in the absence of additional oxidizing reagents. *ACS Catal.* **5**, 3216–3224 (2015).

[23] Kargar, H. et al. Green and efficient removal of sulfides using oxo-peroxo tungsten(VI)-MIL-101(Cr) nanoreactor as heterogeneous recyclable catalyst. *Inorganica Chim. Acta* **545**, 121274 (2023).

[24] Bahadori, M. et al. Task-specific ionic liquid functionalized–MIL–101(Cr) as a heterogeneous and efficient catalyst for the cycloaddition of CO_2 with epoxides under solvent free conditions. *ACS Sustainable Chem. Eng.* **7**, 3962–3973 (2019).

[25] Batra, M. S., Dwivedi, R. & Prasad, R. Recent developments in heterogeneous catalyzed epoxidation of Styrene to styrene oxide. *ChemistrySelect* **4**, 11636–11673 (2019).

[26] Badawy, A. A.-B. Multiple roles of haem in cystathionine β-synthase activity: implications for hemin and other therapies of acute hepatic porphyria. *Biosci. Rep.* **41**, BSR20210935 (2021).

[27] Wang, Q., Wang, K., Tan, X., Li, Z. & Wang, H. Immunomodulatory role of metalloproteases in cancers: Current progress and future trends. *Front. Immunol.* **13**, 1064033 (2022).

[28] Huang, Y., Ren, J. & Qu, X. Nanozymes: classification, catalytic mechanisms, activity regulation, and applications. *Chem. Rev.* **119**, 4357–4412 (2019).

[29] Xu, D., Wu, L., Yao, H. & Zhao, L. Catalase-like nanozymes: classification, catalytic mechanisms, and their applications. *Small* **18**, 2203400 (2022).

[30] Brandes, R. P., Weissmann, N. & Schröder, K. NADPH oxidases in cardiovascular disease. *Free Radic. Biol. Med.* **49**, 687–706 (2010).

[31] Couto, N., Wood, J. & Barber, J. The role of glutathione reductase and related enzymes on cellular redox homoeostasis network. *Free Radic. Biol. Med.* **95**, 27–42 (2016).

[32] Proshlyakov, D. A., Farrugia, M. A., Proshlyakov, Y. D. & Hausinger, R. P. Iron-containing ureases. *Coord. Chem. Rev.* **448**, 214190 (2021).

[33] Wang, M. et al. Recent advances in glucose-oxidase-based nanocomposites for tumor therapy. *Small* **15**, 1903895 (2019).

[34] Ji, H. et al. Hydrogenase as the basis for green hydrogen production and utilization. *J. Energy Chem.* **85**, 348–362 (2023).

[35] Orywal, K. & Szmitkowski, M. Alcohol dehydrogenase and aldehyde dehydrogenase in malignant neoplasms. *Clin. Exp. Med.* **17**, 131–139 (2017).

[36] Golding, A.-L. & Dong, Z. Hydrogen production by nitrogenase as a potential crop rotation benefit. *Environ. Chem. Lett.* **8**, 101–121 (2010).

[37] Jiao, S., Li, F., Yu, H. & Shen, Z. Advances in acrylamide bioproduction catalyzed with Rhodococcus cells harboring nitrile hydratase. *Appl. Microbiol. Biotechnol.* **104**, 1001–1012 (2020).

[38] Choi, J.-M., Han, S.-S. & Kim, H.-S. Industrial applications of enzyme biocatalysis: Current status and future aspects. *Biotechnol. Adv.* **33**, 1443–1454 (2015).

[39] Nam, K. H. Glucose isomerase: functions, structures, and applications. *Appl. Sci.* **12**, 428 (2022).

[40]Ansari, S. A. & Husain, Q. Potential applications of enzymes immobilized on/in nano materials: A review. *Biotechnol. Adv.* **30**, 512–523 (2012).

[41] Sharma, S. K. & Leblanc, R. M. Biosensors based on β-galactosidase enzyme: Recent advances and perspectives. *Anal. Biochem.* **535**, 1–11 (2017).

[42] Jancsó, Z., Hegyi, E. & Sahin-Tóth, M. Chymotrypsin reduces the severity of secretagogue-induced pancreatitis in mice. *Gastroenterology* **155**, 1017–1021 (2018).

[43] Antony, N., Balachandran, S. & Mohanan, P. V. Immobilization of diastase α-amylase on nano zinc oxide. *Food Chem.* **211**, 624–630 (2016).

[44] Sherman, M. R., Saifer, M. G. P. & Perez-Ruiz, F. PEG-uricase in the management of treatment-resistant gout and hyperuricemia. *Adv. Drug Deliv. Rev.* **60**, 59–68 (2008).

[45] Husain, Q. Peroxidase mediated decolorization and remediation of wastewater containing industrial dyes: a review. *Rev. Environ. Sci. Biotechnol.* **9**, 117–140 (2010).

[46] Yoshimoto, M. & Walde, P. Immobilized carbonic anhydrase: preparation, characteristics and biotechnological applications. *World. J. Microbiol. Biotechnol.* **34**, 151 (2018).

[47] Gurung, N., Ray, S., Bose, S. & Rai, V. A Broader View: Microbial enzymes and their relevance in industries, Medicine, and Beyond. *BioMed Res. Int.* **2013**, 1–18 (2013).

[48] Dolor, A. & Szoka, F. C. Digesting a path forward: The utility of collagenase tumor treatment for improved drug delivery. *Mol. Pharmaceutics* **15**, 2069–2083 (2018).

[49] Barzkar, N., Sohail, M., Tamadoni Jahromi, S., Nahavandi, R. & Khodadadi, M. Marine microbial L-glutaminase: from pharmaceutical to food industry. *Appl. Microbiol. Biotechnol.* **105**, 4453–4466 (2021).

[50] Jiang, L. et al. Recent insights into the prognostic and therapeutic applications of lysozymes. *Front. Pharmacol.* **12**, 767642 (2021).

[51] Schwartz, L., Cohen, A., Thomas, J. & Spencer, J. The immunomodulatory and antimicrobial properties of the vertebrate ribonuclease A Superfamily. *Vaccines* **6**, 76 (2018).

[52] Banerjee, A., Chisti, Y. & Banerjee, U. C. Streptokinase—a clinically useful thrombolytic agent. *Biotechnol. Adv.* **22**, 287–307 (2004).

[53] Shah, D. & Mital, K. The role of trypsin:Chymotrypsin in tissue repair. *Adv. Ther.* **35**, 31–42 (2018).

[54] Masucci, M. T., Minopoli, M., Di Carluccio, G., Motti, M. L. & Carriero, M. V. Therapeutic strategies targeting urokinase and its receptor in cancer. *Cancers* **14**, 498 (2022).

[55] Srirangan, K. et al. Biotechnological advances on Penicillin G acylase: Pharmaceutical implications, unique expression mechanism and production strategies. *Biotechnol. Adv.* **31**, 1319–1332 (2013).

[56] Pérez-Ros, P., Navarro-Flores, E., Julián-Rochina, I., Martínez-Arnau, F. M. & Cauli, O. Changes in salivary amylase and glucose in diabetes: A scoping review. *Diagnostics* **11**, 453 (2021).

[57] Jiang, H. & Zhang, X. Acetylcholinesterase and apoptosis: A novel perspective for an old enzyme.

FEBS J. **275**, 612–617 (2008).

[58] Geldenhuys, W. J., Lin, L., Darvesh, A. S. & Sadana, P. Emerging strategies of targeting lipoprotein lipase for metabolic and cardiovascular diseases. *Drug Discov. Today* **22**, 352–365 (2017).

[59] Damin, B. I. S., Kovalski, F. C., Fischer, J., Piccin, J. S. & Dettmer, A. Challenges and perspectives of the β-galactosidase enzyme. *Appl. Microbiol. Biotechnol.* **105**, 5281–5298 (2021).

[60] Chong, Y., Liu, Q. & Ge, C. Advances in oxidase-mimicking nanozymes: Classification, activity regulation and biomedical applications. *Nano Today* **37**, 101076 (2021).

[61] Cao, C. et al. Biomedicine meets nanozyme catalytic chemistry. *Coord. Chem. Rev.* **491**, 215245 (2023).

[62] Wu, J. et al. Ligand-dependent activity engineering of glutathione peroxidase-mimicking MIL-47(V) metal-organic framework nanozyme for therapy. *Angew. Chem. Int. Ed.* **60**, 1227 – 1234 (2021).

[63] Luo, Q. et al. Stabilizing ultrasmall ceria-cluster nanozyme for antibacterial and antibiofouling applications. *Small* **18**, 2107401 (2022).

[64] Peng, G., González, V., Vázquez, E., Lundberg, J. O. & Fadeel, B. Two-dimensional molybdenum disulfide nanosheets evoke nitric oxide-dependent antibacterial effects. *Nanoscale* **15**, 17409–17421 (2023).

[65] Chen, Y., Chen, T., Wu, X. & Yang, G. Oxygen vacancy-engineered PEGylated MoO_{3-x} nanoparticles with superior sulfite oxidase mimetic activity for vitamin B1 detection. *Small* **15**, 1903153 (2019).

[66] Cao, C. et al. Starvation, ferroptosis, and prodrug therapy synergistically enabled by a cytochrome c oxidase like nanozyme. *Adv. Mater.* **34**, 2203236 (2022).

[67] Qian, X. et al. Nitric oxide producing artificial enzymes based on metalloporphyrins. *Mater. Today Chem.* **23**, 100743 (2022).

[68] Zhe, Y. et al. Ascorbate oxidase-like nanozyme with high specificity for inhibition of cancer cell proliferation and online electrochemical DOPAC monitoring. *Biosens. Bioelectron.* **220**, 114893 (2023).

[69] Wu, Y., Chen, W., Wang, C. & Xing, D. Overview of nanozymes with phosphatase-like activity. *Biosens. Bioelectron.* **237**, 115470 (2023).

[70] Yu, M. et al. Intimate coupling of photocatalysis and biodegradation for wastewater treatment: Mechanisms, recent advances and environmental applications. *Water Res.* **175**, 115673 (2020).

[71] Kamkaew, A. *et al.* BODIPY dyes in photodynamic therapy. *Chem. Soc. Rev.* **42**, 77–88 (2013).

[72] Boyjoo, Y., Sun, H., Liu, J., Pareek, V. K. & Wang, S. A review on photocatalysis for air treatment: From catalyst development to reactor design. *Chem. Eng. J.* **310**, 537–559 (2017).

[73] Yang, H., Liu, R., Xu, Y., Qian, L. & Dai, Z. Photosensitizer nanoparticles boost photodynamic therapy for pancreatic cancer treatment. *Nano-Micro Lett.* **13**, 35 (2021).

[74] Nguyen, V.-H. et al. Photocatalytic NO_x abatement: Recent advances and emerging trends in the development of photocatalysts. *J. Clean. Prod.* **270**, 121912 (2020).

[75] Li, S. et al. Light-switchable yolk–mesoporous shell UCNPs@$MgSiO_3$ for nitric oxide-evoked multidrug resistance reversal in cancer therapy. *ACS Appl. Mater. Interfaces* **12**, 30066–30076 (2020).

[76] Huang, L. et al. Visible-light-driven photocatalytic oxidation of H_2S by boron-doped TiO_2/LDH Heterojunction: Synthesis, performance, and reaction mechanism. *Chem. Eng. J.* **448**, 137607 (2022).

[77] Yang, Z. et al. Photothermo-promoted nanocatalysis combined with H_2S-mediated respiration inhibition for efficient cancer therapy. *Adv. Funct. Mater.* **31**, 2007991 (2021).

[78] Abdi, J., Sisi, A. J., Hadipoor, M. & Khataee, A. State of the art on the ultrasonic-assisted removal of environmental pollutants using metal-organic frameworks. *J. Hazard. Mater.* **424**, 127558 (2022).

[79] Costley, D. *et al.* Treating cancer with sonodynamic therapy: A review. *Int. J. Hyperthermia* **31**, 107–117 (2015).

[80] Remya, N. & Lin, J.-G. Current status of microwave application in wastewater treatment—A review. *Chem. Eng. J.* **166**, 797–813 (2011).

[81] Yu, M. et al. Magnetic bimetallic heterointerface nanomissiles with enhanced microwave absorption for microwave thermal/dynamics therapy of breast cancer. *ACS Nano* **18**, 3636–3650 (2024).

[82] Chia, X. & Pumera, M. Characteristics and performance of two-dimensional materials for electrocatalysis. *Nat. Catal.* **1**, 909–921 (2018).

[83] Lu, Z. et al. Porous Pt nanospheres incorporated with GO_x to enable synergistic oxygen-inductive starvation/electrodynamic tumor therapy. *Adv. Sci.* **7**, 2001223 (2020).

[84] Prabhu, P., Jose, V. & Lee, J. Heterostructured catalysts for electrocatalytic and photocatalytic carbon dioxide reduction. *Adv. Funct. Mater.* **30**, 1910768 (2020).

[85] Yan, H. et al. Emerging delivery strategies of carbon monoxide for therapeutic applications: from CO gas to CO releasing nanomaterials. *Small* **15**, 1904382 (2019).

[86] Pan, J. et al. Electrocatalytic hydrogen evolution reaction related to nanochannel materials. *Small Struct.* **2**, 2100076 (2021).

[87] Wu, Y. et al. *In vivo* X-ray triggered catalysis of H_2 generation for cancer synergistic gas radiotherapy. *Angew. Chem. Int. Ed.* **60**, 12868–12875 (2021).

[88] Xu, Y. et al. A critical review of research progress for metal alloy materials in hydrogen evolution and oxygen evolution reaction. *Environ. Sci. Pollut. Res.* **30**, 11302–11320 (2022).

[89] Zu, Y., Wang, Z., Yao, H. & Yan, L. Oxygen-generating biocatalytic nanomaterials for tumor hypoxia relief in cancer radiotherapy. *J. Mater. Chem. B* **11**, 3071–3088 (2023).

[90] Ouedraogo, A. S. & Bhoi, P. R. Recent progress of metals supported catalysts for hydrodeoxygenation of biomass derived pyrolysis oil. *J. Clean. Prod.* **253**, 119957 (2020).

[91] Pham, C. Q. et al. Production of hydrogen and value-added carbon materials by catalytic methane decomposition: a review. *Environ. Chem. Lett.* **20**, 2339–2359 (2022).

[92] Onishi, N. & Himeda, Y. Homogeneous catalysts for CO_2 hydrogenation to methanol and methanol dehydrogenation to hydrogen generation. *Coord. Chem. Rev.* **472**, 214767 (2022).
